# Microwave-Assisted Extraction of Polyphenols from Bitter Orange Industrial Waste and Identification of the Main Compounds

**DOI:** 10.3390/life13091864

**Published:** 2023-09-04

**Authors:** Juan F. García-Martín, Chao-Hui Feng, Nelson-Manuel Domínguez-Fernández, Paloma Álvarez-Mateos

**Affiliations:** 1 Departamento de Ingeniería Química, Facultad de Química, Universidad de Sevilla, C/Profesor García González, 1, 41012 Seville, Spainpalvarez@us.es (P.Á.-M.); 2 School of Regional Innovation and Social Design Engineering, Faculty of Engineering, Kitami Institute of Technology, 165 Koen-cho, Kitami 090-8507, Japan; feng.chaohui@mail.kitami-it.ac.jp; 3 RIKEN Centre for Advanced Photonics, RIKEN, 519-1399 Aramaki-Aoba, Sendai 980-0845, Japan

**Keywords:** flavonoids, microwave-assisted extraction, neohesperidin, orange waste

## Abstract

In this work, the extraction of phenolic compounds from orange waste (OW) obtained after the industrial extraction of neohesperidin from bitter oranges (Seville oranges) was assayed by microwave-assisted extraction (MAE) and Soxhlet extraction (SE). The extraction agents were ethanol and acetone. For SE, aqueous solutions of both extraction agents were used at 50%, 75%, and 100% (*v*/*v*). For MAE, a design of experiments was applied to determine the conditions that maximize the extraction yield. The independent variables were temperature (from 20 to 75 °C), process time (between 10 and 20 min), and percentage of extraction agent (*v*/*v*) in the extraction solution (50%, 75%, and 100%). Following that, the extracts were analyzed by ultra-high-performance liquid chromatography to identify the main phenolic compounds extracted. Results showed that 50% (*v*/*v*) ethanol or acetone was the extraction agent concentration that maximized the extraction yield for both SE and MAE, with the yields of MAE being higher than those of SE. Thus, the highest extraction yields on a dry basis achieved for MAE were 16.7 g/100 OW for 50% acetone, 75 °C, and 15 min, and 20.2 g/100 OW for 50% ethanol, 75 °C, and 10.8 min, respectively. Finally, the main phenolic compounds found in the orange waste were naringin, hesperidin, neohesperidin, and naringenin (i.e., flavonoids).

## 1. Introduction

Flavonoids are one of the most important groups of dietary phenolic compounds, which have antioxidant, anticancer, anti-inflammatory, anti-allergic, and antimicrobial properties and can provide cardiovascular health benefits [1]. Moreover, they have important antiviral properties. For example, naringenin, in combination with cycloheximide, protects cells against damage induced by tumor necrosis factor α [2] and possesses a high potential for the treatment of different types of cancer [3]. Flavonoids have been associated with cardiovascular health benefits. Some studies suggest that certain flavonoids, such as quercetin and catechins, may help improve heart health by reducing oxidative stress, improving blood vessel function, and lowering blood pressure. Flavonoid-rich foods, such as berries and cocoa, have been linked to a reduced risk of heart disease in observational studies [4]. As with heart disease, flavonoids’ potential benefits for stroke risk reduction are linked to their antioxidant and anti-inflammatory properties, which may help to protect the brain’s blood vessels and reduce the risk of ischemic stroke. Some studies have suggested a potential association between flavonoid intake and a reduced risk of stroke [5,6,7]. In addition, hesperidin, herbacetin, rhoifolin, and pectolinarin have been reported to bind the key protease in the functioning of the SARS-CoV virus, thus blocking its enzymatic activity [8,9,10]. For that reason, flavonoids are regarded as a starting point for therapeutics against COVID-19 [11]. However, flavonoids are not synthesized by the human body because they are phytochemicals [12].

On the other hand, flavonoids such as naringin, hesperidin, and neohesperidin are high-added-value products with a high commercial price [13,14]. Numerous companies are therefore interested in optimizing the extraction of these compounds from natural sources.

Oranges are one of the main sources of flavonoids, hence the importance of extracting flavonoids from them. Moreover, oranges have been highlighted as a potential source of other high-added-value products such as carotenoids, dietary fiber, carbohydrates, sugars, and pectin [15,16]. The global production of oranges for the year 2022/2023 is estimated to be 47.3 million tons [17]. The orange industry, including harvesting, transport, processing, storage, and distribution, is responsible for millions of jobs around the world and there is much ongoing research on improving orange processing and extending the shelf life of fruit [18,19]. Bitter oranges, also known as Seville oranges, come from the *Citrus aurantium* tree. Because of their neohesperidin content, their taste is bitter and therefore they are mainly used for medicines and cosmetics at an industrial scale [13].

Phenolic compounds from orange waste are commonly extracted by solvent extraction methods. The orange waste is soaked in a solvent for a defined period of time. Afterward, the mixture is centrifuged, and then the supernatant is filtered, which is subsequently concentrated through the evaporation of the solvent [16]. Among the conventional extraction techniques, Soxhlet extraction (SE) is the most commonly used. SE is a continuous and exhaustive extraction method, which can lead to a high yield of target compounds, including essential oils and flavonoids from orange peels. Notwithstanding, the main drawbacks of SE are its long extraction time (several hours), high operational temperature, huge solvent consumption, and its need to resort to an evaporation stage to concentrate the extracts [13,20]. Assisted extraction techniques are considered to overcome these constraints. Among them, ultrasound-assisted extraction (UAE) and microwave-assisted extraction (MAE) stand out. Ultrasound waves can provoke cavitation bubbles, which enhance mass transfer and improve the contact between the solvent and the OW, resulting in higher extraction efficiency, while microwave energy can rapidly heat the solvent and OW, leading to faster extraction compared to traditional methods. In spite of increasing extraction yields and decreasing solvent consumption and extraction time, these techniques have limitations. For instance, microwave energy can rupture cell walls and heat the water in cytoplasmic contents, as well as decompose the phytochemical compounds in flavonoids; hence, MAE might not be suitable when the objective is the biological activity of flavonoids [16,21]. On the other hand, an increase in ultrasound frequency results in free radical generation, which in turn leads to undesirable changes in the extracted compounds [22]. For the extraction of bioactive compounds, MAE has been reported to be more suitable than UAE because MAE requires less extraction time and provides higher extraction yields [23]. For example, the recovery of total polyphenols from *C. sinensis* peels, using aqueous acetone in different concentrations as extraction solvent, obtained 356.75, 305.41, and 301.27 kg/ton∙h using MAE, UAE, and conventional solvent extraction, respectively [24]. In the extraction of phenolic acids from mandarin peels (*C. reticulata*) using 80% (*v*/*v*) aqueous ethanol solution as extraction agent, results indicated that MAE provided the highest content of ferulic acid (0.239 g/100 g) when compared with UAE (0.235 g/100 g) and conventional extraction in a rotary shaker at room temperature (0.205 g/100 g DW) [25]. Hence, MAE was selected as the extraction technique for the present work. A comprehensive classification of the flavonoid extraction techniques along with their fundamentals can be found elsewhere [23].

With regard to the extraction solvent, methanol, ethanol, acetone, and ethyl acetate are the most commonly used solvents for flavonoid extraction [26]. Combinations of these solvents and aqueous solutions of them are also used according to the sample type (peel waste or pulp waste) and the water content in the sample. Methanol has been pointed out to be the most effective solvent for extracting flavonoid compounds of low molecular weight, while aqueous acetone solutions are preferred for the extraction of high-molecular-weight flavonoid compounds [26].

In a previous work, flavonoids were extracted from sweet orange peels, and from ripe and unripe bitter oranges, by Soxhlet extraction using different concentrations of ethanol and methanol aqueous solutions (50:50, 80:20, and 100:0 solvent-to-water ratios (% *v*/*v*)) [13]. It was concluded that ethanol was the most suitable solvent to extract flavonoids from *C. aurantium* oranges, reaching a maximum extraction yield of 15.5 wt.% with pure ethanol. In the present work, flavonoids were extracted from an industrial waste using MAE. This waste is obtained in the extraction of neohesperidin from Seville bitter oranges at the industrial facilities of a company. With regard to the extraction solvent, not only aqueous ethanol solutions were used, because of the results obtained in the previous work [13], but also aqueous acetone solutions, since acetone has been reported as the most suitable solvent for polyphenols in MAE because of its wide polarity range [27,28]. Temperature and extraction time were, along with the solvent-to-water ratio, the operational parameters studied. A Box–Behnken experimental design was performed to reduce the number of experiments to be carried out and to obtain the conditions that would maximize the extraction yield with both solvents. The Box–Behnken design was chosen because it has fewer design points than central composite design for the same number of factors (in this case 3), thus reducing the cost and time of the experiments. This is because the Box–Behnken design avoids all the corner points and the star points, so that as many center points as used in central composite design are not needed because points on the outside are closer to the middle. Finally, SE with the same solvent-to-water ratios used in the MAE was performed to compare both extraction techniques.

## 2. Materials and Methods

### 2.1. Raw Materials

The orange waste (OW) used throughout this research was supplied by Bordas S.A. (Dos Hermanas, Spain). This industry extracts, through a confidential procedure, neohesperidin from Seville bitter oranges (*C. aurantium* L.). The extraction yield of this flavonoid in Bordas S.A. facilities is not 100%, so the orange waste should still contain neohesperidin. Once at the laboratory, the OW was dried at 40 °C and immediately ground and sieved through a 1 mm mesh.

### 2.2. Soxhlet Extraction (SE)

Roughly 30 g of dried OW (*W*_1_) was placed in a cellulose cartridge along with the required solvent volume to cover the Soxhlet body. It was then refluxed for 5 h. Afterward, the solvent was separated from the extract using a rotary evaporator (Heindolph Hei-VAP Core, Schwabach, Germany). The Soxhlet extract was dried in an oven at 40 °C and then weighed (*W*_2_). The extraction yield of SE (*ηs_E_*) was calculated as follows:

(1) $$\eta_{SE}\left(\%\right)=\frac{W_{2}}{W_{1}}\times 100$$

As solvents, aqueous solutions of ethanol and acetone were used in solvent-to-water ratios (% *v*/*v*) of 100:0, 75:25, and 50:50. The dried extracts obtained were stored at 4 °C until used for ultra-high-performance liquid chromatography (UHPLC) analysis. The experiments were carried out in duplicate.

### 2.3. Microwave-Assisted Extraction (MAE)

MAE of polyphenols from OW was carried out in a microwave digester (Milestone Ethos One, Sorisole, Italy) at 500 W power using aqueous ethanol or aqueous acetone solutions as solvent. Approximately 1 g of dried OW (*W*_1_) was mixed with 20 mL of solvent (i.e., 1/20 *m*/*v* sample-to-solvent ratio) and placed in teflon vessels which were closed before starting the experiments in the microwave digester. The heating time to reach the desired temperature was set to 7 min, while the time to cool down the samples was set to 10 min. The obtained extracts were filtered in cellulose filter paper, dried in an oven at 40 °C, weighed (*W*_2_), and stored at 4 °C until they were analyzed using HPLC. A Box–Behnken experimental design was carried out using Design Expert 13 software (Stat-Ease, Inc., Minneapolis, MN, USA). The independent variables were process temperature (T), extraction time (t), and solvent-to-water ratio (S:W), as illustrated in Table 1, while the dependent variable was the extraction yield (*η_MAE_*). Similarly to SE, the extraction yield of MAE was calculated as follows:

(2) $$\eta_{MAE}(\%)=\frac{W_{2}}{W_{1}}\times 100$$

As a result of the design of experiments, fifteen MAE were run (Table 2), each of them in triplicate.

### 2.4. Determination of Phenolic Compounds

Tentative analysis of the phenolic compounds present in the extracts was carried out using a binary UHPLC Dionex UltiMate 3000 RS coupled to a quadrupole-orbitrap QExactive hybrid mass spectrometer (Thermo Scientific, Waltham, MA, USA) equipped with heated electrospray ionization probe and an Acquity UPLC BEH C18 column (100 × 2.1 mm, 130 Ǻ, 1.7 μm) (Waters, Milford, CT, USA).

Dried extracts were suspended in a 50% (*v*/*v*) methanol/0.1% (*v*/*v*) formic acid solution, filtered using a 0.2 μm pore size nylon filter, and then 5 μL was injected in the UHPLC system. The working conditions of the UHPLC system were 40 °C temperature and 0.5 mL/min flow rate. The elution took place using a mixture of (A) water with (B) 0.1% (*v*/*v*) methanol solution, both mixed with 0.1% (*v*/*v*) formic acid. The elution gradient was 95% A and 5% B for 0–10 min, 100% B for 10–12 min, and 95% A and 5% B for 12–15 min. The analysis process was controlled using Xcalibur software (Thermo Fisher Scientific, Waltham, MA, USA).

The identification of phenolic compounds was made by comparing the retention times and the exact masses of pseudo-molecular ions and their fragment ions with the database of the Quan Browser tool of the XCalibur 4.3 software. TraceFinder software version 5.1 (Thermo Fisher Scientific, Waltham, MA, USA) was used for data treatment. To limit the number of phenolic compounds, it was established that the area of the compound must have a value greater than 1, that the probability of the compound must be greater than 40%, and that the compound must be present in a significant number of samples [29,30].

For the tentative quantification of each identified polyphenol, the individual extraction yields (*η_P_*) were calculated as follows:

(3) $$\eta_{P}\left(\%\right)=\frac{{A\times W}_{2}}{W_{1}}\times 100$$

where (*W*_2_) is the mass of the extract, (*W*_1_) stands for the mass of the sample, and *A* is the relative area of the phenolic compound identified by UHPLC.

### 2.5. Model Evaluation

The performance of the models was assessed using the adjusted R^2^ and the predicted R^2^. The adjusted R^2^ (for the number of parameters in the model in relation to the number of points in the design) is a measure of the variation of the mean according to the model, while the predicted R^2^ is a measure of the model’s performance in predicting a response value. The selected criterion was to find the model that maximizes the adjusted R^2^. The adjusted R^2^ and the predicted R^2^ must not differ by 0.20 or more from each other in order to be in reasonable agreement. If they do, that may suggest a problem with either the data or the model.

## 3. Results

### 3.1. Soxhlet Extraction

The extraction yields for SE using both ethanol and acetone aqueous solutions are shown in Table 3. While the effect of the ethanol-to-water ratio on the *η_SE_* was not clear, a decrease in the acetone-to-water ratio enhanced the SE.

With regard to the UHPLC analysis, the main compounds identified were flavonoids, more specifically, hesperidin, naringenin, neohesperidin, and naringin, although small quantities of protocatechuic acid, chlorogenic acid (3-O-caffeoylquinic acid), caffeic acid, umbelliferone, gallic acid, and 4-hydroxybenzoic acid were found as well. The extraction yields of these compounds were very low, as illustrated in Table 4.

### 3.2. Microwave-Assisted Extraction

The extraction yields for MAE using both acetone and ethanol aqueous solutions as solvent are illustrated in Table 5. For this extraction technique, it was found that decreasing the percentage of acetone or ethanol in the extraction solvent resulted in an increase in the *η_MAE_*. Thus, the highest extraction yields were found using 50% (*v*/*v*) acetone or ethanol solutions.

With regard to the extraction yields of the polyphenols identified by UHPLC, Table 6, Table 7 and Table 8 shows their *η_P_* for solvent-to-water ratios of 50, 75, and 100% (*v*/*v*), respectively.

### 3.3. Surface Response Methodology (RSM) for MAE

The Box–Behnken designs for MAE using both acetone and ethanol aqueous solutions as solvent achieved *η_MAE_* ranging between 1.18 and 16.68 wt.% for acetone and from 0.76 to 16.09 wt.% for ethanol.

When developing a quadratic model that takes into account the effect of the three independent variables on the response, it was found that both the linear interactions and the quadratic interactions were significant (*p* < 0.05) when using aqueous acetone solutions, leading to the following equation:

*η_MAE_* (wt.%) = −9.74 + 0.55 T + 0.71 t + 0.19 S:W − 0.0037 T × t − 0.0023 T × S:W + 0.00056 t × S:W − 0.0024 T^2^ − 0.017 t^2^ + 0.0022 S:W^2^ (4)

**Figure 1:**
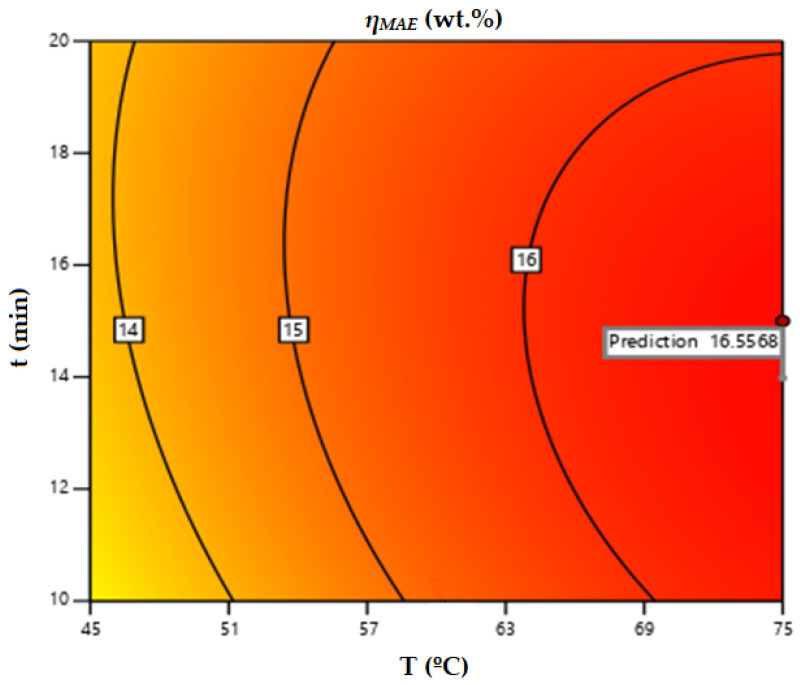
Contour surface obtained for microwave-assisted extraction yield using acetone as solvent.

According to the model, and using Equation (4) for the range of temperature (°C), time (min), and acetone/water (% *v*/*v*) percentages tested, a microwave extraction yield of 16.55% could be achieved under the conditions of 75 °C, 14 min, and 50% (*v*/*v*) S:W. This theoretical *η_MAE_* does not improve the actual one achieved experimentally and used in the design of experiments (16.67%), obtained at 75 °C, 15 min, and 50% (*v*/*v*) S:W. For an acetone:water solution at 50% (*v*/*v*), the contour plot obtained for the microwave extraction yield as a function of temperature and extraction time would be as depicted in Figure 1.

With regard to the use of ethanol as the solvent for MAE, it was found that only the linear interactions of the three independent variables have an influence on the model (*p* < 0.05), rendering the following equation for the yield of the microwave-assisted extraction:

*η_MAE_* (wt.%) = 23.043 + 0.065 T + 0.202 t − 0.291 S:W (5)

As illustrated in Table 9, the statistics of this model were worse than those of the MAE using acetone as solvent. Even so, the predicted R^2^ (0.7838) was in reasonable agreement with the adjusted R^2^ (0.8551); that is, the difference was less than 0.2. According to the model for MAE using ethanol as the extraction solvent, the application of Equation (5) in the range of T, t, and S:W assayed leads to maximum microwave extraction yield of 20.21 wt.% under the conditions of 75 °C temperature, 10.8 min extraction time, and a 50% (*v*/*v*) ethanol:water solution.

**Figure 2:**
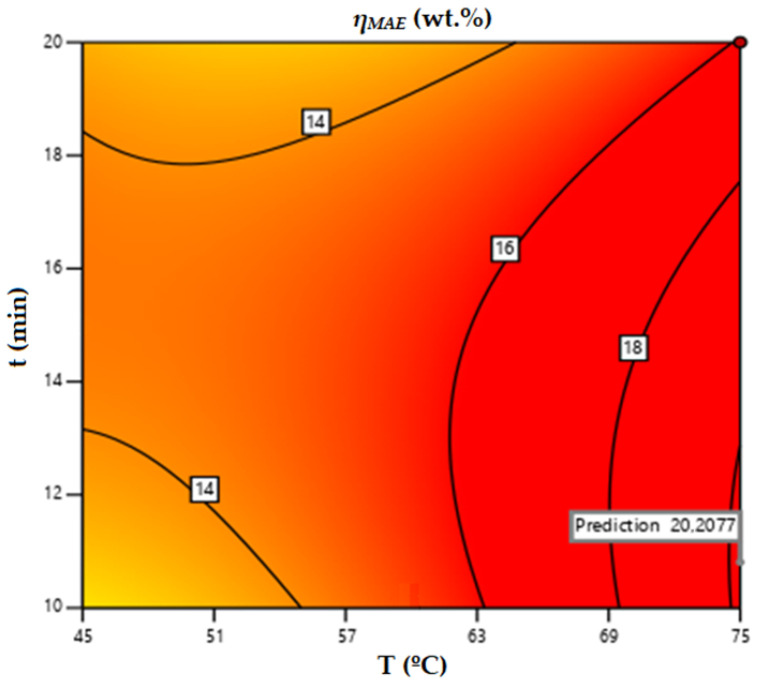
Contour surface obtained for microwave-assisted extraction yield using ethanol as solvent.

For an ethanol:water solution at 50% (*v*/*v*), the contour plot obtained for the microwave extraction yield as a function of temperature and process time would be as depicted in Figure 2, where the maximum *η_MAE_* is highlighted.

## 4. Discussion

The use of 50 and 75% (*v*/*v*) aqueous acetone solutions maximized *η_SE_* while the highest *η_MAE_* was found using ethanol/water solutions. It is well known that acetone is a relatively polar solvent and has the ability to dissolve a wide range of organic compounds. It is particularly effective in extracting polar and semi-polar compounds, including many of the constituents found in orange peels. The combination of acetone and water allows for a better partitioning and extraction of a broader spectrum of compounds from the orange peels. Since SE takes a long time, using a solvent such as aqueous acetone can improve efficiency by enhancing the solubility and extraction of target compounds from the orange peels. As for MAE, it employs microwave radiation to heat the solvent quickly. The rapid heating, combined with the unique solvent properties of ethanol/water, enhances the extraction process by promoting better diffusion and dissolution of the target compounds from the orange peels [31]. In a study on the extraction of bioactive compounds from willow gentian, the authors explained that ethanol has a lower dielectric constant than water, and pointed out that a higher content of ethanol in the mixture reduces the dipole moment of the solvent and thus reduces the absorption of microwave radiation [31].

Response surface methodology has been employed to model and optimize the extraction of phenolic compounds from various orange by-products by evaluating the effects of multiple factors and their interactions on response variables [24,32,33,34]. In the findings of other authors, the optimum extraction of polyphenols from *C. sinensis* orange pomace (byproduct of the orange juice industry) by vacuum MAE was predicted by RSM to be 37,667 mg/kg under the following conditions: microwave power = 6000 W, water-to-orange-pomace ratio (L/kg) = 26.1, and extraction time = 120 min [35]. The maximum extraction yield of total polyphenols in lab-scale batch MAE from *C. sinensis* peels using 50% (*v*/*v*) aqueous acetone solution as extraction agent, as predicted by RSM, was 12.10 ± 0.15 mg/g at 500 W and a solvent-to-solid ratio of 25 mL/g for 120 s [24]. Those authors found that increasing the microwave power over 500 W (the microwave power also used in the present work) led to a decrease in the TPC extraction yield. The use of strong microwave powers leads to an increase in the temperature, which has a negative influence on thermo-labile compounds [34]. Those authors also found that by extending the extraction time under these conditions, the TPC yield decreased [24]. As for UAE of phenolic compounds from *C. sinensis* orange peels, RSM was applied to investigate the extraction conditions, resulting in the identification of optimal parameters including an ultrasound extraction time of 44 min, a temperature of 50 °C, and 57.7% (*v*/*v*) ethanol as extraction solvent [33]. Under these optimized conditions, the total phenolic content was reported to be 292.16 µg catechol/g, while the total flavonoid content was measured at 191.14 µg catechol/g [33]. These yields were slightly higher than the ones obtained in the present work in the MAE of phenolic compounds using ethanol as extraction solvent, but it should be taken into account that the orange waste used here comes from the neohesperidin extraction from bitter oranges (*C. aurantium*) at an industrial scale.

**Figure 3:**
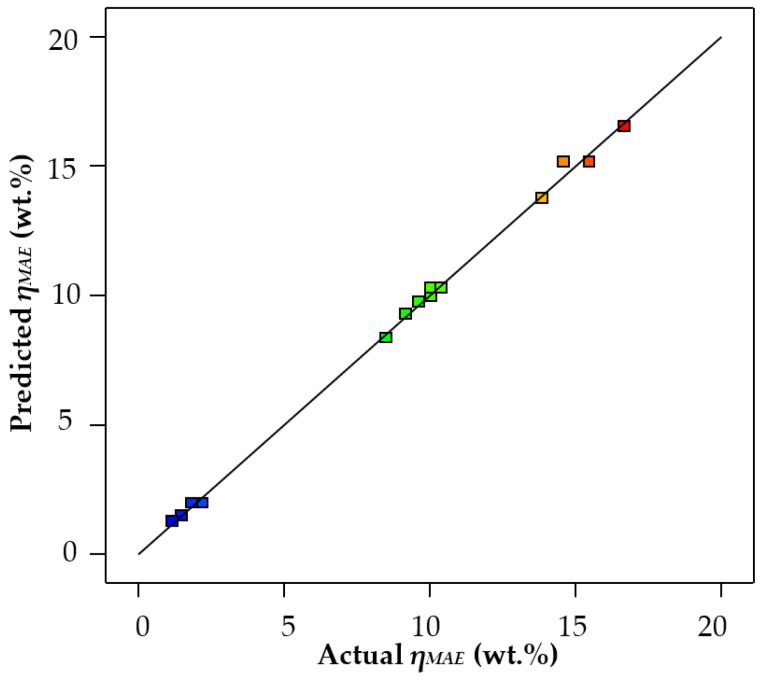
Microwave-assisted extraction yields predicted using the Design Expert 13 software vs. actual yields, using acetone as extraction solvent.

**Figure 4:**
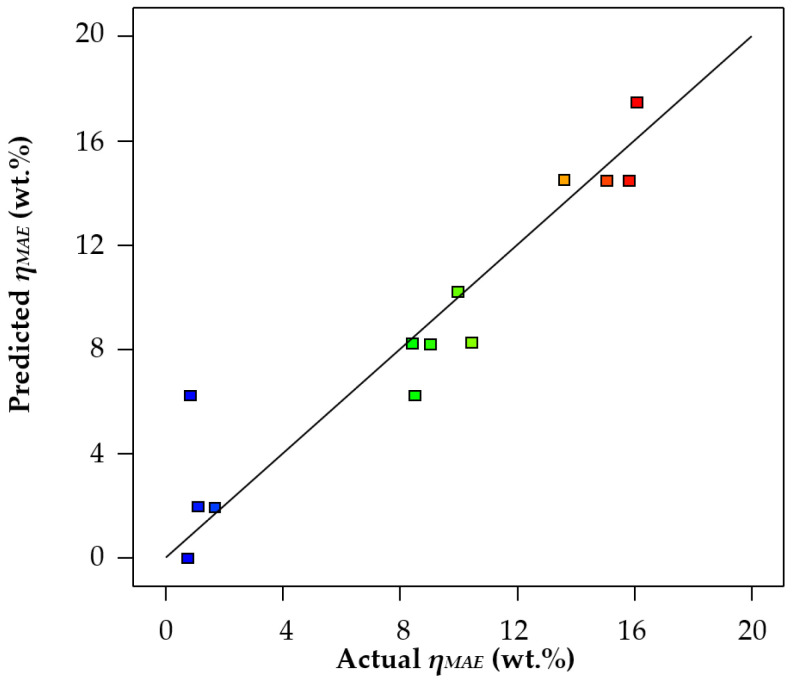
Microwave-assisted extraction yields predicted using the Design Expert 13 software vs. actual yields, using ethanol as extraction solvent.

The goodness of the models obtained (Equations (4) and (5)) is illustrated in Figure 3 and Figure 4, where the actual extraction yields obtained in the laboratory are plotted against those predicted by the models.

As can be seen, the data of the actual values match the values predicted by the model as obtained with acetone as extraction solvent. On the contrary, the model for ethanol showed some lack of precision, so that some predicted yields did not match very well with the actual yields. That is to say, the results for *η_MAE_* obtained using ethanol were varied and unstable, which reduced the precision of the fit of the regression model, so that the results were distributed on the regression line. This is also consistent with the observation that the R^2^ for acetone (0.9983) was higher than that for ethanol (0.8861) (Table 9). This could be because ethanol is not as specific as acetone for the extraction of phenolic compounds. Ethanol is reported to be used for the extraction of essential oils in oranges [36] and, in general, for the SE of oil from food materials [37]. By contrast, acetone as an extraction solvent avoids problems related to pectin, such as its clotting properties, and allows the use of much lower temperatures. Thus, extraction with acetone has been pointed out as a more efficient and more reproducible extraction method [38]. Hence, the results obtained for ethanol might not be as reliable as those achieved in the MAE with acetone.

From Table 4, Table 6, Table 7 and Table 8 it can be seen that hesperidin, naringenin, neohesperidin, and naringin were the main phenolic compounds (all of them flavonoids) found in the extracts. This is in agreement with the flavonoids found by other authors in this type of citrus. For instance, naringin and neohesperidin were found to be the major polyphenols in bitter orange peels, while narirutin and hesperidin were the main polyphenols in sweet orange peels [39]. Hesperidin and narirutin have been reported as the most predominant flavanones in orange fruit [40]. In addition, the flavonoids in sweet orange (*C. sinensis*) peel extracts have also been detected using Fourier transform infrared (FTIR) spectroscopy (500 cm^−1^ to 4000 cm^−1^) and terahertz spectroscopy (0.5–9 THz) [14]. Both techniques showed a similar fingerprint for hesperidin, indicating that hesperidin was the main phenolic compound in orange peel extracts. Of note is that flavonoid detection using terahertz spectroscopy provides a rapid and simplified analysis procedure. Also, it is easy to distinguish the spectra of hesperidin and naringin in comparison with the time-consuming and complex sample pre-treatment required when using HPLC. However, terahertz spectroscopy works only as a qualitative technique, being unable to quantify the amount of each flavonoid, which limits its application.

Hesperidin content has been found to be higher in peels than in juice or seeds, and it is probably responsible for fruit coloration [16]. Since the industrial orange waste used in this work contains orange peel, this fact could account for the high hesperidin content found in it. It is of major importance because the inhibitory effect of hesperidin against the development of neurodegenerative diseases has been confirmed by clinical evidence [41]. This industrial orange waste could be converted into a high-added-value by-product. The small concentration of naringenin found in OW is also remarkable due to its potential for the treatment of different diseases [2,3] and antidiabetic properties [42]. Notwithstanding, the antidiabetic potential of naringenin has yet to be demonstrated in a clinical setting, i.e., more human studies are needed, specifically, more studies in individuals suffering from insulin resistance, obesity, and diabetes mellitus Type 2 [42].

While neohesperidin was the main flavonoid obtained by SE, naringin was the main compound in the extracts from MAE. This could be due to the fact that the orange waste comes from a process of neohesperidin extraction at an industrial scale, which could cause the waste to have non-extracted neohesperidin easily accessible for solvent extraction. Since the *η_SE_* values were much lower than those of *η_MAE_*, it could simply indicate that neohesperidin was easier to extract by both methods, although the main phenolic compound in the orange waste was naringin. Notwithstanding, it must be taken into account that the concentrations of the different phenolic compounds in these tables were calculated from relative areas, not by using internal standards for each compound. In any case, they show that the industrial waste obtained after neohesperidin extraction at an industrial scale still contains significant amounts of neohesperidin, along with high concentrations of other flavonoids (mainly naringin) and other phenolic acids.

## 5. Conclusions

Based on the results obtained, MAE achieves higher yields in shorter extraction times than SE with the same S:W, using both aqueous ethanol and acetone solutions.

The use of ethanol as the extraction solvent, for both SE and MAE, did not result in changes in the composition of the extracts and extraction yields. The highest experimental *η_MAE_* (15.83 wt.%) was obtained using ethanol as the extraction solvent at a temperature of 60 °C, 20 min extraction time, and S:W of 50:50% (*v*/*v*), while the highest *η_SE_* (7.58 wt.%) was obtained with acetone at a 50:50% (*v*/*v*) solvent-to-water ratio. With regard to the results obtained using acetone as solvent, the highest extraction yields were achieved with S:W of 50:50 (% *v*/*v*).

According to the results obtained by UHPLC analysis, the main polyphenolic compounds identified in the industrial waste from *C. aurantium* L. oranges were flavonoids. More specifically, hesperidin, neohesperidin, naringenin, and naringin were detected, along with other phenolic acids in lower concentrations, among which naringin was the one with the greatest content, followed by neohesperidin. This is of major importance because flavonoids are used in a variety of food and beverage products, as well as in dietary supplements.

Overall, the orange waste from neohesperidin extraction at an industrial scale should be regarded as a valuable by-product from which high-added-value products (mainly flavonoids) can be extracted in large quantities. The main limitations of this research are that the study was conducted on a small scale and that the results may not be generalizable to other types of orange waste. Future research could involve larger sample sizes, encompassing various types of orange waste from different regions and industries and employing multiple extraction methods. Researchers should transparently acknowledge these limitations in study reports to facilitate a better understanding and interpretation of the findings by the scientific community and industry stakeholders.

## Figures and Tables

**Table 1:** Design of experiments for MAE.

Factor	Name	Units	Minimum	Maximum	Coded Low	Coded High	Mean
A	T	°C	45	75	−1 ↔ 45	+1 ↔ 75	60
B	t	min	10	20	−1 ↔ 10	+1 ↔ 20	15
C	S:W	% *v*/*v*	50	100	−1 ↔ 50	+1 ↔ 100	75

**Table 2:** Number of experiments and conditions (in coded values) for MAE.

Run	A	B	C
1	−1	0	−1
2	0	0	0
3	0	−1	−1
4	1	−1	0
5	−1	0	1
6	0	1	−1
7	1	0	−1
8	0	0	0
9	−1	−1	0
10	−1	1	0
11	0	−1	1
12	0	1	1
13	0	0	0
14	1	1	0
15	1	0	1

**Table 3:** Extraction yields for Soxhlet extraction (*η_SE_*) at different solvent-to-water ratios (S:W).

Ethanol		Acetone	
S:W (% *v*/*v*)	*η_SE_* (wt.%)	S:W (% *v*/*v*)	*η_SE_* (wt.%)
100	5.47 ± 0.18	100	0.51 ± 0.05
75	4.46 ± 0.11	75	6.45 ± 0.09
50	4.56 ± 0.16	50	7.58 ± 0.14

**Table 4:** Extraction yields of different polyphenols for SE at different solvent-to-water ratios (S:W).

**Solvent**	Acetone	Ethanol	Acetone	Ethanol	Acetone	Ethanol
**S:W (% *v*/*v*)**	50	50	75	75	100	100
**Hesperidin (wt.%)**	0.69	0.47	0.55	0.41	0.02	0.13
**Neohesperidin (wt.%)**	1.29	0.92	1.15	0.93	0.16	1.28
**Naringenin (wt.%)**	1.06	0.43	0.99	0.58	0.01	0.54
**Naringin (wt.%)**	2.03	1.47	1.81	1.48	0.03	0.31
**Other acids (wt.%)**	1.55	1.00	1.43	1.18	0.27	2.92

**Table 5:** Extraction yields for MAE (*η_MAE_*) using acetone-water and ethanol-water solutions as extraction solvent.

S:W (% *v*/*v*)	Run	T (°C)	t (min)	*η_MAE_* (wt.%)	
				Acetone	Ethanol
50	1	45	15	13.85 ± 0.03	13.62 ± 0.15
	3	60	10	14.61 ± 0.04	15.07 ± 0.09
	6	60	20	15.48 ± 0.05	15.83 ± 0.12
	7	75	15	16.68 ± 0.04	16.09 ± 0.11
75	2	60	15	10.41 ± 0.03	8.43 ± 0.13
	4	20	10	10.05 ± 0.02	9.06 ± 0.15
	9	45	10	8.51 ± 0.01	8.51 ± 0.11
	10	45	20	9.18 ± 0.05	10.45 ± 0.16
	14	75	20	9.62 ± 0.03	9.98 ± 0.08
100	5	45	15	1.84 ± 0.03	0.76 ± 0.12
	11	60	10	1.50 ± 0.02	0.84 ± 0.06
	12	60	20	2.22 ± 0.04	1.12 ± 0.07
	15	75	15	1.18 ± 0.04	1.69 ± 0.09

**Table 6:** Polyphenol yields for MAE using 50% (*v*/*v*) S:W.

**Solvent**	Acetone	Ethanol	Acetone	Ethanol	Acetone	Ethanol	Acetone	Ethanol
**T (°C)**	45	45	60	60	60	60	75	75
**t (min)**	15	15	10	10	20	20	15	15
**Hesperidin (wt.%)**	1.09	0.69	1.35	0.93	1.40	0.75	2.08	1.70
**Neohesperidin (wt.%)**	3.07	3.26	3.49	3.32	3.71	2.23	3.82	3.32
**Naringenin (wt.%)**	1.98	1.57	1.79	1.80	1.96	1.19	2.04	1.78
**Naringin (wt.%)**	4.80	5.09	5.34	5.11	5.73	3.53	6.32	5.18
**Other acids (wt.%)**	1.99	1.52	1.97	1.91	1.74	1.11	1.90	1.90

**Table 7:** Polyphenol yields for MAE using 75% (*v*/*v*) S:W.

**Solvent**	Acetone	Ethanol	Acetone	Ethanol	Acetone	Ethanol	Acetone	Ethanol	Acetone	Ethanol
**T (°C)**	60	60	75	75	45	45	45	45	45	45
**t (min)**	15	15	10	10	10	10	15	15	10	10
**Hesperidin (wt.%)**	0.88	0.55	0.86	0.65	0.59	0.47	0.83	0.60	0.92	0.76
**Neohesperidin (wt.%)**	2.16	1.69	1.72	1.77	1.46	1.66	1.97	2.04	1.73	1.99
**Naringenin (wt.%)**	1.68	1.41	1.44	1.44	1.25	1.44	1.31	1.92	1.84	1.18
**Naringin (wt.%)**	3.32	2.69	2.76	2.82	2.29	2.58	3.10	3.22	2.73	3.23
**Other acids (wt.%)**	1.74	1.39	1.57	1.46	1.37	1.51	1.43	2.02	1.81	1.32

**Table 8:** Polyphenol yields for MAE using 100% (*v*/*v*) S:W.

**Solvent**	Acetone	Ethanol	Acetone	Ethanol	Acetone	Ethanol	Acetone	Ethanol
**T (°C)**	45	45	60	60	60	60	75	75
**t (min)**	15	15	10	10	20	20	15	15
**Hesperidin (wt.%)**	0.12	0.05	0.21	0.07	0.17	0.07	0.16	0.11
**Neohesperidin (wt.%)**	0.21	0.08	0.30	0.13	0.33	0.14	0.23	0.24
**Naringenin (wt.%)**	0.10	0.12	0.14	0.16	0.43	0.22	0.14	0.38
**Naringin (wt.%)**	0.31	0.13	0.48	0.21	0.50	0.23	0.37	0.39
**Other acids (wt.%)**	0.06	0.12	0.11	0.21	0.40	0.25	0.13	0.47

**Table 9:** Factorial design statistics for microwave-assisted extraction.

**Solvent**	Acetone	Ethanol
**Standard deviation**	0.36	2.05
**Mean value (wt.%)**	9.04	8.55
**Coefficient of variation (%)**	3.99	23.98
**R^2^**	0.9983	0.8861
**Adjusted R^2^**	0.9952	0.8551
**Predicted R^2^**	0.9723	0.7838
